# Quadriceps Tendon Autografts Are Associated With Increased Short-term Reoperation Rates for Extension Deficit or Pain and Inferior Patient-Reported Outcomes Compared With Hamstring Tendon Autografts: An Analysis of 5653 Cases of Primary Anterior Cruciate Ligament Reconstruction

**DOI:** 10.1177/03635465261465107

**Published:** 2026-08-03

**Authors:** Carolina Kekki, Riccardo Cristiani, Anders Stålman, Christoffer von Essen

**Affiliations:** † Department of Molecular Medicine and Surgery, Stockholm Sports Trauma Research Center, Karolinska Institutet, Stockholm, Sweden; ‡ Capio Artro Clinic, FIFA Medical Centre of Excellence, Stockholm, Sweden; Investigation performed at Capio Artro Clinic and the Stockholm Sports Trauma Research Center, Karolinska Institutet, Stockholm, Sweden

**Keywords:** anterior cruciate ligament reconstruction, graft choice, failure rate, nonrevision reoperation, KOOS

## Abstract

**Background:**

Anterior cruciate ligament reconstruction (ACLR) rates are rising, and quadriceps tendon (QT) autografts have gained popularity in recent years. Although QT autografts show objective and subjective outcomes comparable to hamstring tendon (HT) and bone–patellar tendon–bone (BPTB) autografts, nonrevision reoperations across graft types and their impact on patient-reported outcome measure (PROM) scores remain poorly studied.

**Purpose:**

To compare nonrevision reoperation rates and PROM scores between primary ACLR using HT, BPTB, and QT autografts at 2 years’ follow-up.

**Study Design:**

Cohort study; Level of evidence, 3.

**Methods:**

In this retrospective cohort study, 5653 primary ACLR procedures (2015-2022) were categorized by autograft type. Reoperations for extension deficit or pain, meniscal injuries, or cartilage injuries within 2 years were identified through medical records. Revision ACLR and graft failure were not included. Patient-reported outcomes were assessed preoperatively and at 2 years postoperatively using the Knee Injury and Osteoarthritis Outcome Score (KOOS). The minimal important change, patient acceptable symptom state, and treatment failure were evaluated using the KOOS_4_. Subgroup analysis examined associations between graft diameter, sex, and reoperations for extension deficit or pain.

**Results:**

The QT group had a higher overall reoperation rate (19.2%) than the HT (8.9%) and BPTB (6.9%) groups (*P* < .001), driven by extension deficit or pain (11.9% vs 5.5% and 4.7%, respectively; *P* < .001). QT grafts remained independently associated with reoperations for extension deficit or pain after adjusting for confounders (odds ratio [OR], 1.96 [95% confidence interval (CI), 1.44-2.67]; *P* < .001). The QT group demonstrated lower odds of achieving the minimal important change than the HT (OR, 0.56 [95% CI, 0.41-0.77]; *P* < .001) and BPTB (OR, 0.48 [95% CI, 0.32-0.72]; *P* < .001) groups. The QT and BPTB groups showed higher odds of experiencing treatment failure (OR, 1.98 [95% CI, 1.21-3.23]; *P* = .01 and OR, 2.00 [95% CI, 1.10-3.63]; *P* = .02) and lower odds of achieving the patient acceptable symptom state (OR, 0.65 [95% CI, 0.49-0.85]; *P* = .002 and OR, 0.57 [95% CI, 0.41-0.79]; *P* < .001) than the HT group. On sex-stratified analysis, female patients demonstrated higher reoperation rates for extension deficit or pain than male patients (7.3% vs 5.5%, respectively; *P* = .01), and female patients receiving larger grafts (9.5-12.0 mm) demonstrated 72% higher odds of having this outcome (OR, 1.72 [95% CI, 1.14-2.56]; *P* = .01).

**Conclusion:**

QT autografts were associated with significantly higher reoperation rates for extension deficit or pain and inferior PROM scores compared with HT autografts at 2 years after primary ACLR.

Anterior cruciate ligament (ACL) injuries are among the most common knee injuries, particularly affecting young and physically active patients. The number of ACL reconstruction (ACLR) procedures performed annually has increased in recent years.^14,30^ For patients aiming to return to high-demand activities involving cutting, pivoting, or jumping, ACLR remains the recommended treatment strategy.^6^ However, despite extensive research, the optimal autograft choice for primary ACLR is still debated.

Historically, hamstring tendon (HT) and bone–patellar tendon–bone (BPTB) autografts have been the most commonly used grafts for primary ACLR.^1,30^ However, in recent years, the quadriceps tendon (QT) autograft has gained popularity because of its favorable biomechanical properties and low donor site morbidity.^30^ Several studies have reported that QT autografts yield comparable objective and subjective outcomes to HT and BPTB autografts in primary ACLR.^3,23,33^ Consequently, the use of QT autografts has increased from approximately 1% to nearly 10% over the past decade, while HT autograft usage has declined.^1,29,30^

Despite this growing interest, most previous comparisons of autograft types have relied on small cohorts and primarily focused on graft failure and revision ACLR. Far fewer studies have evaluated nonrevision reoperations—including reoperations for extension deficit or pain, meniscal injuries, and cartilage injuries—which may affect early recovery, reflect graft-related complications, and influence patient satisfaction. Moreover, it remains unclear whether differences in early reoperation patterns between graft types translate into meaningful variations in patient-reported outcome measure (PROM) scores. A comprehensive assessment of nonrevision reoperations and PROM scores across the 3 principal autograft types is therefore needed to better inform graft selection and perioperative decision-making.

The primary aim of the current study was to compare the 2-year incidence of nonrevision reoperations (for extension deficit or pain, meniscal injuries, or cartilage injuries) after ACLR performed with HT, BPTB, or QT autografts. The secondary aim was to compare PROM scores across graft types at 2 years after primary ACLR. Furthermore, subgroup analysis compared graft diameter and the reoperation rate for extension deficit or pain between sexes. It was hypothesized that QT autografts would be associated with higher reoperation rates for extension deficit or pain, owing to their anatomic and biomechanical characteristics, and with inferior patient-reported outcomes compared with HT and BPTB autografts.

## Methods

Ethical approval was obtained from the regional ethics committee of Karolinska Institutet (No. 2016/1613-31/32). All procedures and protocols adhered to the Declaration of Helsinki.

### Patients

This retrospective cohort study included all patients who underwent primary ACLR between January 2015 and December 2022 at Capio Artro Clinic. Patients were identified through the clinic's database and allocated into 3 groups based on the type of autograft used at index surgery: HT, BPTB, or QT. Because this study was designed to evaluate nonrevision reoperations after primary ACLR, patients who underwent revision ACLR or had evidence of ACL graft failure within the 2-year follow-up period were excluded. The inclusion of these cases would fundamentally alter the study population, potentially confounding analysis of nonrevision reoperations. Additional exclusion criteria were concomitant ligament injuries requiring surgical treatment, including injuries to the medial collateral ligament, lateral collateral ligament, posterior cruciate ligament, and posterolateral corner as well as combinations of these injuries (combined ligament injuries).

### Data Collection

Baseline patient data were retrieved from the clinic's database including sex, age at primary ACLR, laterality of injury, cartilage or meniscal injuries, and laterality of meniscal injury. Intraoperative data were retrieved from the operative report including graft type and size as well as type of meniscal treatment (repair or resection). Subsequent surgery within 2 years of primary ACLR was identified and categorized using the NOMESCO (Nordic Medico-Statistical Committee) classification of surgical procedures.^18^ Operative reports were reviewed, and each reoperation was assigned to ≥1 prespecified category: extension deficit or pain (including cyclops lesions, arthrofibrosis, and notch impingement), meniscal injury, or cartilage injury. For patients with multiple reoperations, only the first event was used in primary event–based analysis to avoid clustering bias, whereas when >1 procedure was performed during a single reoperation, all procedures were recorded, allowing a patient to be included in >1 reoperation subgroup. Other reoperations, such as hardware removal, were not included in this study. For analysis of patient-reported outcomes, each patient contributed one observation only. Patient-reported outcomes were assessed using the Knee Injury and Osteoarthritis Outcome Score (KOOS),^22^ obtained from the Swedish Knee Ligament Registry.^30^

### Patient-Reported Outcome Measures

All patients were asked to complete the KOOS preoperatively and at 2 years postoperatively. The KOOS includes 42 questions divided into 5 subscales: Pain, Symptoms, Activities of Daily Living, Sport and Recreation, and Quality of Life. Each question is answered on a 5-point Likert scale, and the score for each subscale is transformed into a subscore ranging from 0 to 100, with a higher score indicating a better outcome.^22^

This study utilized the KOOS_4_, calculated as the average of the Pain, Symptoms, Sport and Recreation, and Quality of Life subscales. The Activities of Daily Living subscale is excluded from the KOOS_4_ because of its susceptibility to ceiling effects in young, active populations undergoing ACLR.^7^ The KOOS_4_ was used to derive the minimal important change (MIC), patient acceptable symptom state (PASS), and treatment failure (TF). The MIC was defined as the smallest clinically relevant change in the KOOS_4_ score.^31^ The PASS identified patients with satisfactory knee function in the operated knee and was assessed by a single yes/no question: “Taking into account all the activities you have during your daily life, your level of pain, and also your activity limitations and participation restrictions, do you consider the current state of your knee satisfactory?”^17^ TF identified patients with unsatisfactory postoperative knee function. In the current study, threshold values for the MIC, PASS, and TF according to the KOOS_4_, previously defined by Roos et al^21^ through anchor-based methods, were used.^11,12^ Threshold values for the MIC, the PASS, and TF were 9, 79, and 42, respectively.

### Surgical Technique and Rehabilitation

All patients underwent arthroscopic single-bundle ACLR using HT, BPTB, or QT autografts. The semitendinosus tendon was used for HT autografts, prepared as a tripled or quadrupled graft, supplemented with the gracilis tendon if the diameter was <8 mm. BPTB autografts included the central third of the patellar tendon with 2 bone blocks. QT autografts were harvested using a standard technique. In most cases, a full-thickness graft was obtained; however, the exact harvest technique (full vs partial thickness) could vary slightly depending on surgeon preference. Most QT autografts included a patellar bone block. Femoral fixation used the Endobutton device (Smith & Nephew) until 2017, and thereafter, the TightRope device (Arthrex) was used. Tibial fixation was achieved with Ethibond sutures (Ethicon) tied over an AO screw (AO Foundation), with interference screws used in a minority of cases. Meniscal tears in the posterior horn or corpus were repaired with an all-inside technique using Fast-Fix (Smith & Nephew), with meniscal tears in the anterior horn repaired with an outside-in technique using PDS 0 (Ethicon) and meniscal root tears repaired with a transtibial pull-out technique.

All patients were advised to follow a standardized rehabilitation protocol. Full weightbearing was allowed immediately postoperatively. Those with concomitant meniscal repair used a hinged knee brace for 6 weeks, with a limitation in flexion of 0° to 30° during weeks 0 to 2, 0° to 60° during weeks 2 to 4, and 0° to 90° during weeks 4 to 6. Return to sports was permitted no earlier than 9 months postoperatively.

### Statistical Analysis

Statistical analysis was performed using RStudio (Version 4.3.1; Posit). Descriptive statistics were reported as means and standard deviations for normally distributed continuous variables, medians and interquartile ranges for nonnormally distributed continuous variables, and counts and percentages for categorical variables. Normality was assessed using the Shapiro-Wilk test. Graft diameter and age were categorized into 3 and 4 subgroups, respectively. Comparisons between graft groups were performed using the chi-square test for categorical variables, the Student *t* test for continuous variables, and the Fisher exact test for counts expected to be <5. Multivariable logistic regression analyses were performed with reoperation for meniscal injuries, cartilage injuries, or extension deficit or pain, MIC, PASS, and TF as dependent variables in separate models; and age, sex, graft type, graft diameter, laterality of injury, concomitant meniscal surgery (repair or resection), and cartilage injury as independent variables to adjust for potential confounding. Patients missing preoperative or 2-year KOOS scores were excluded from multivariable analyses. Subgroup analysis compared graft diameter and reoperation rates for extension deficit or pain between male and female patients, and logistic regression assessed the association between graft diameter and reoperation rates for extension deficit or pain stratified by sex. Sensitivity analysis excluding female patients with the largest graft size (9.5-12.0 mm) from the main analysis was performed (see the Appendix, available in the online version of this article). A *P* value < .05 was considered statistically significant.

## Results

### Patient Characteristics

**Figure 1.**
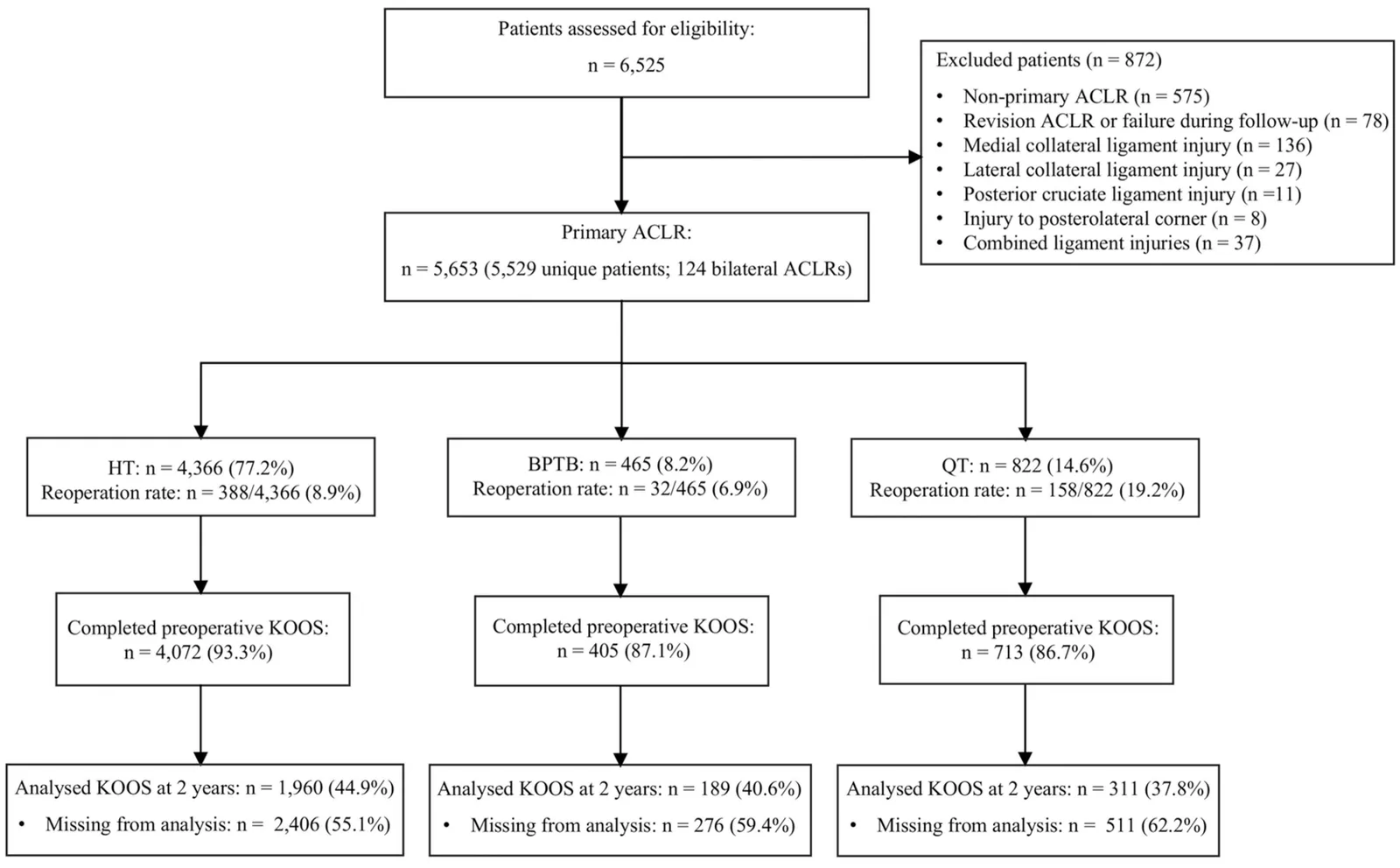
Overview of patient selection and Knee Injury and Osteoarthritis Outcome Score (KOOS) response rates across graft types. Combined ligament injuries represent injuries involving ≥2 of the following: medial collateral ligament, lateral collateral ligament, posterior cruciate ligament, or posterolateral corner. ACLR, anterior cruciate ligament reconstruction; BPTB, bone–patellar tendon–bone; HT, hamstring tendon; QT, quadriceps tendon.

A total of 5653 primary ACLR procedures in 5529 unique patients were included in the study: 4366 (77.2%) involved an HT autograft, 465 (8.2%) involved a BPTB autograft, and 822 (14.6%) involved a QT autograft. KOOS scores at 2-year follow-up were obtained for 1960 cases (44.9%) in the HT group, 189 (40.6%) in the BPTB group, and 311 (37.8%) in the QT group (Figure 1). The QT group was significantly younger, included a lower proportion of female patients, and had higher rates of concomitant meniscal and cartilage injuries than the HT and BPTB groups. Patient characteristics at baseline for the 3 graft groups are reported in Table 1.

**Table 1:** Patient Characteristics at Baseline*^*a*^*

	HT (n = 4366)	BPTB (n = 465)	QT (n = 822)	*P* Value
Age at primary ACLR, median (IQR), y	28.0 (20.0-40.0)	27.0 (20.0-38.0)	26.0 (19.0-35.0)	<.001
Sex				<.001
Female	2182 (50.0)	205 (44.1)	346 (42.1)	
Male	2184 (50.0)	260 (55.9)	476 (57.9)	
Laterality of injury				.03
Right	2279 (52.2)	232 (49.9)	388 (47.2)	
Left	2087 (47.8)	233 (50.1)	434 (52.8)	
Graft diameter, mm				<.001
6.0-8.0	1786 (40.9)	6 (1.3)	18 (2.2)	
8.5-9.0	2011 (46.1)	128 (27.5)	242 (29.4)	
9.5-12.0	569 (13.0)	331 (71.2)	562 (68.4)	
Cartilage injury	748 (17.1)	84 (18.1)	185 (22.5)	.001
Meniscal injury	2096 (48.0)	176 (37.8)	443 (53.9)	<.001
Medial	1332 (30.5)	80 (17.2)	245 (29.8)	<.001
Lateral	1115 (25.5)	129 (27.7)	292 (35.5)	<.001
Medial meniscal resection	559 (12.8)	30 (6.5)	78 (9.5)	<.001
Medial meniscal repair	731 (16.7)	46 (9.9)	166 (20.2)	<.001
Lateral meniscal resection	566 (13.0)	79 (17.0)	126 (15.3)	.02
Lateral meniscal repair	511 (11.7)	47 (10.1)	172 (20.9)	<.001
Time from ACLR to reoperation, median (IQR), d	301 (201-425)	266 (131-460)	317 (201-457)	.70

a Data are reported as n (%) unless otherwise specified. ACLR, anterior cruciate ligament reconstruction; BPTB, bone–patellar tendon–bone; HT, hamstring tendon; IQR, interquartile range; QT, quadriceps tendon.

### Reoperations

Within 2 years of surgery, the QT group demonstrated a higher overall reoperation rate compared with the HT and BPTB groups. Extension deficit or pain was the most common indication for a reoperation, occurring in 11.9% of the QT group versus 5.5% of the HT group and 4.7% of the BPTB group (*P* < .001). Reoperation rates for meniscal and cartilage injuries were similar across graft types (Table 2).

**Table 2:** Reoperation Rates Within 2 Years of ACLR*^*a*^*

	HT (n = 4366)	BPTB (n = 465)	QT (n = 822)	*P* Value
Extension deficit or pain	240 (5.5)	22 (4.7)	98 (11.9)	<.001
Meniscal injuries	108 (2.5)	9 (1.9)	42 (5.1)	.92
Cartilage injuries	40 (0.9)	1 (0.2)	18 (2.2)	.31
All reoperations	388 (8.9)	32 (6.9)	158 (19.2)	<.001

a Data are reported as n (%). ACLR, anterior cruciate ligament reconstruction; BPTB, bone–patellar tendon–bone; HT, hamstring tendon; QT, quadriceps tendon.

After adjusting for age, sex, graft type, graft diameter, laterality of injury, concomitant meniscal surgery, and cartilage injury, QT autografts remained independently associated with significantly higher odds of a reoperation for extension deficit or pain (odds ratio [OR], 1.96 [95% confidence interval (CI), 1.44-2.67]; *P* < .001) compared with HT autografts. Male sex was associated with lower odds of a reoperation for extension deficit or pain (OR, 0.63 [95% CI, 0.50-0.80]; *P* < .001), and concomitant meniscal repair increased the odds of this outcome (OR, 2.12 [95% CI, 1.68-2.69]; *P* < .001) (Table 3).

**Table 3:** Multivariable Logistic Regression Analysis for Reoperations*^*a*^*

	Meniscal Injury		Cartilage Injury		Extension Deficit or Pain	
	OR (95% CI)	*P* Value	OR (95% CI)	*P* Value	OR (95% CI)	*P* Value
Age, y						
20-30	Reference		Reference		Reference	
<20	1.07 (0.65-1.74)	.80	0.78 (0.38-1.64)	.52	0.80 (0.60-1.07)	.13
31-40	0.93 (0.55-1.59)	.80	0.83 (0.39-1.78)	.63	0.97 (0.71-1.30)	.82
>40	1.10 (0.63-1.93)	.74	0.94 (0.44-2.01)	.88	0.79 (0.58-1.09)	.15
Sex						
Female	Reference		Reference		Reference	
Male	1.34 (0.88-2.03)	.17	1.20 (0.66-2.18)	.56	0.63 (0.50-0.80)	<.001
Graft type						
BPTB vs HT	1.19 (0.50-2.84)	.70	0.22 (0.03-1.72)	.15	0.80 (0.49-1.31)	.38
QT vs HT	0.91 (0.52-1.57)	.73	0.91 (0.43-1.93)	.81	1.96 (1.44-2.67)	<.001
QT vs BPTB	0.76 (0.32-1.85)	.55	4.12 (0.53-32.16)	.18	0.86 (0.42-1.74)	.68
Graft diameter, mm						
6.0-8.0	Reference		Reference		Reference	
8.5-9.0	1.37 (0.83-2.27)	.21	1.58 (0.74-3.33)	.24	1.14 (0.86-1.51)	.36
9.5-12.0	1.02 (0.51-2.04)	.95	1.93 (0.73-5.10)	.18	1.32 (0.91-1.91)	.15
Laterality of injury						
Right	Reference		Reference		Reference	
Left	0.94 (0.64-1.37)	.75	1.56 (0.90-2.70)	.11	0.98 (0.79-1.22)	.89
Meniscal surgery						
No surgery	Reference		Reference		Reference	
Repair	5.58 (3.57-8.73)	<.001	1.17 (0.63-2.19)	.61	2.12 (1.68-2.69)	<.001
Resection	1.34 (0.65-2.76)	.43	1.98 (0.91-4.29)	.08	0.84 (0.59-1.18)	.31
Cartilage injury						
No	Reference		Reference		Reference	
Yes	1.10 (0.69-1.76)	.69	1.35 (0.70-2.58)	.37	0.94 (0.70-1.26)	.68

a BPTB, bone–patellar tendon–bone; HT, hamstring tendon; OR, odds ratio; QT, quadriceps tendon.

### Subgroup Analysis by Sex

Male patients received grafts of a significantly larger diameter than female patients (median, 9.0 vs 8.5 mm, respectively; *P* < .001). Female patients experienced significantly more reoperations for extension deficit or pain than male patients (7.3% vs 5.5%, respectively; *P* = .01) (Table 4). On logistic regression stratified by sex, female patients receiving larger grafts (9.5-12.0 mm) had 72% increased odds of a reoperation for extension deficit or pain. An increased graft diameter was associated with higher odds of a reoperation for extension deficit or pain in both sexes; however, statistical significance was reached only for the largest graft diameter in female patients (Table 5).

**Table 4:** Subgroup Analysis by Sex*^*a*^*

	Male (n = 2920)	Female (n = 2733)	*P* Value
Graft diameter, median (IQR), mm	9.0 (8.5-9.5)	8.5 (8.0-9.0)	<.001
Graft diameter, mm			<.001
6.0-8.0	501 (17.2)	1289 (47.2)	
8.5-9.0	1319 (45.2)	1070 (39.2)	
9.5-12.0	1100 (37.6)	374 (13.6)	
Reoperation for extension deficit or pain	160 (5.5)	200 (7.3)	.01

a Data are reported as n (%) unless otherwise specified. IQR, interquartile range.

**Table 5:** Association Between Sex, Graft Diameter, and Extension Deficit or Pain*^*a*^*

Sex	Graft Diameter, mm	OR (95% CI)	*P* Value
Male	6.0-8.0	Reference	Reference
	8.5-9.0	1.12 (0.69-1.90)	.66
	9.5-12.0	1.70 (0.96-2.82)	.07
Female	6.0-8.0	Reference	Reference
	8.5-9.0	1.25 (0.91-1.73)	.16
	9.5-12.0	1.72 (1.14-2.56)	.01

a OR, odds ratio.

### PROM Scores

**Figure 2.**
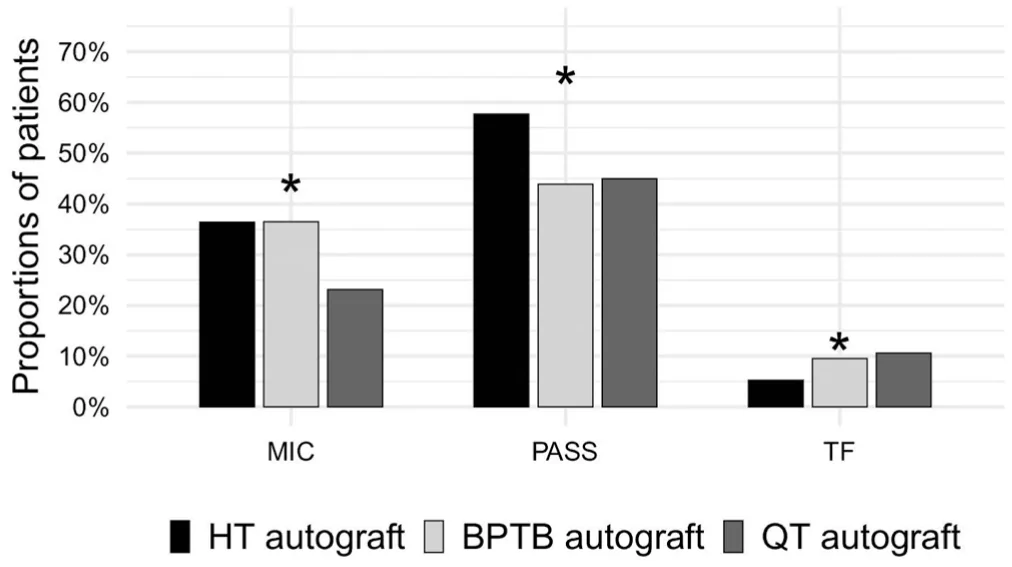
Proportion of patients achieving the minimal important change (MIC), achieving the patient acceptable symptom state (PASS), and experiencing treatment failure (TF) according to the KOOS_4_ across graft types at 2-year follow-up. BPTB, bone–patellar tendon–bone; HT, hamstring tendon; QT, quadriceps tendon. *Statistically significant.

At 2-year follow-up, a significantly greater proportion of patients reached the PASS threshold in the HT group (57.7%) than in the BPTB (43.9%) and QT (45.0%) groups (*P* < .001). The HT group demonstrated significantly fewer TF (5.3%) than the BPTB (9.5%) and QT (10.6%) groups (*P* < .001). A significantly smaller proportion of patients in the QT group achieved the MIC (23.2%) compared with the HT (36.4%) and BPTB (36.5%) groups (*P* < .001) (Figure 2).

Multivariable logistic regression analysis, adjusted for age, sex, graft type, graft diameter, laterality of injury, meniscal surgery, and cartilage injury, demonstrated that QT autografts had significantly lower odds of achieving the MIC than BPTB and HT autografts. Both BPTB and QT autografts had significantly higher odds of experiencing TF and lower odds of achieving the PASS than HT autografts. Patients with concomitant meniscal repair had lower odds of achieving the PASS than those without meniscal surgery. A cartilage injury was associated with lower odds of achieving the MIC. Patients aged <20 years had significantly higher odds of achieving the PASS and decreased odds of experiencing TF, while those aged >40 years showed significantly higher odds of achieving the PASS (Table 6).

**Table 6:** Multivariable Logistic Regression Analysis for PROMs*^*a*^*

	MIC		PASS		TF	
	OR (95% CI)	*P* Value	OR (95% CI)	*P* Value	OR (95% CI)	*P* Value
Age, y						
20-30	Reference		Reference		Reference	
<20	1.07 (0.85-1.35)	.55	1.56 (1.25-1.94)	<.001	0.57 (0.36-0.91)	.02
31-40	1.04 (0.81-1.34)	.77	1.14 (0.89-1.45)	.30	0.95 (0.60-1.51)	.83
>40	1.07 (0.84-1.35)	.60	1.40 (1.11-1.75)	.004	0.88 (0.56-1.37)	.57
Sex						
Female	Reference		Reference		Reference	
Male	1.08 (0.90-1.30)	.40	1.23 (1.03-1.47)	.02	1.17 (0.82-1.68)	.38
Graft type						
BPTB vs HT	1.16 (0.82-1.64)	.40	0.57 (0.41-0.79)	<.001	2.00 (1.10-3.63)	.02
QT vs HT	0.56 (0.41-0.77)	<.001	0.65 (0.49-0.85)	.002	1.98 (1.21-3.23)	.01
QT vs BPTB	0.48 (0.32-0.72)	<.001	1.14 (0.78-1.64)	.50	0.99 (0.53-1.83)	.97
Graft diameter, mm						
6.0-8.0	Reference		Reference		Reference	
8.5-9.0	0.87 (0.71-1.06)	.16	0.90 (0.74-1.09)	.28	1.32 (0.85-2.03)	.21
9.5-12.0	0.79 (0.59-1.06)	.11	0.92 (0.70-1.22)	.57	1.05 (0.58-1.88)	.88
Laterality of injury						
Right	Reference		Reference		Reference	
Left	1.11 (0.94-1.31)	.24	1.08 (0.92-1.27)	.33	0.94 (0.68-1.31)	.73
Meniscal surgery						
No surgery	Reference		Reference		Reference	
Repair	1.41 (1.16-1.71)	<.001	0.74 (0.62-0.90)	.002	1.65 (1.15-2.37)	.01
Resection	1.31 (1.04-1.65)	.02	1.03 (0.82-1.29)	.80	0.84 (0.51-1.41)	.52
Cartilage injury						
No	Reference		Reference		Reference	
Yes	0.79 (0.63-0.99)	.04	0.86 (0.69-1.06)	.17	1.11 (0.73-1.67)	.63

a BPTB, bone–patellar tendon–bone; HT, hamstring tendon; MIC, minimal important change; OR, odds ratio; PASS, patient acceptable symptom state; PROM, patient-reported outcome measure; QT, quadriceps tendon; TF, treatment failure.

## Discussion

The main finding of this study was that patients who underwent primary ACLR with QT autografts had significantly higher rates of reoperations for extension deficit or pain than those with HT autografts. Furthermore, QT autografts yielded inferior PROM scores across all evaluated metrics (MIC, PASS, and TF), whereas HT autografts demonstrated the most favorable results. Subgroup analysis stratified by sex showed that female patients receiving larger grafts (9.5-12.0 mm in diameter) had 72% higher odds of a reoperation for extension deficit or pain than those receiving a smaller graft. These findings add nuance to previous reports suggesting comparable outcomes between QT and other autografts.^3,23,33^

The elevated odds of a reoperation for extension deficit or pain observed in the QT group may partly be attributed to the graft's anatomic and biomechanical characteristics. QT autograft has the largest cross-sectional area among the 3 graft types,^9,32^ and its multilayered structure with interwoven fibers may predispose to mechanical irritation within the intercondylar notch, particularly if the graft is oversized.^26,27^ Unlike HT and BPTB autografts, QT autografts may leave loose collagen fibers exposed, increasing the risk of extension deficit or pain.^25,26^ Additionally, QT grafts are stiffer^9^ and have a more uniform thickness^32^ than HT and BPTB grafts, potentially intensifying mechanical irritation during early rehabilitation. Multivariable logistic regression analysis supported these findings for QT autografts compared with HT autografts after adjusting for potential confounders.

Few prior studies have compared reoperations for extension deficit or pain across graft types, limiting contextual interpretation. Schmücker et al^25^ reported higher rates of cyclops lesions after ACLR with QT autografts (5.0%) than HT autografts (2.4%), although not statistically significant. Other studies have reported short-term reoperation rates for cyclops lesions of 1.8%^5^ and 3.7%^15^ after primary ACLR, although not stratifying by graft type. Reoperation rates for arthrofibrosis after ACLR with QT autografts have been reported as 7.2% at 6 months^8^ and 8.3% at a mean of 57.6 months,^10^ with the latter compared with BPTB (6.0%) and HT (3.3%) autografts. These studies defined a reoperation as a procedure for extension deficit and/or pain, although intraoperative findings varied. Differences in reported reoperation rates may reflect varying definitions, follow-up durations, and indications for a reoperation—for example, one study recommended a reoperation for any degree of extension deficit or pain at 6 months,^8^ potentially inflating reoperation rates. Furthermore, in this study, extension deficit or pain was defined as clinically significant symptoms causing substantial limitations in activities of daily living. Intraoperative findings included cyclops lesions, arthrofibrosis, and notch impingement. The higher reoperation rates observed may therefore in part be explained by this broader definition of indication.

Subgroup analysis stratified by sex showed that male patients received significantly larger grafts than female patients. Previous research has reported larger femoral notch volumes in male patients,^4^ which may accommodate larger grafts. Multivariable logistic regression analysis indicated that male sex was associated with reduced odds of a reoperation for extension deficit or pain, possibly reflecting the generally wider femoral notch in men. Furthermore, female patients receiving larger grafts (9.5-12.0 mm) had 72% higher odds of a reoperation for extension deficit or pain than those receiving smaller grafts. A similar trend was observed in male patients, although not statistically significant. Sensitivity analysis excluding female patients with graft sizes of 9.5 to 12.0 mm showed no significant change in the main results (Table 3), suggesting that this subgroup did not drive the main findings, although its small sample size may have limited its influence. These findings highlight the importance of individualized graft selection and a careful assessment of graft size relative to notch morphology, particularly in female patients.

In terms of PROMs, the QT group demonstrated inferior outcomes across all measures on crude analysis, while the BPTB group showed significantly inferior outcomes in the PASS and TF compared with the HT group. On multivariable logistic regression analysis, the QT group demonstrated significantly lower odds of achieving the MIC compared with both the HT and BPTB groups. In addition, both the QT and BPTB groups showed higher odds of experiencing TF and significantly lower odds of achieving the PASS than the HT group. A systematic review by Sun et al^28^ reported higher odds of achieving the MIC and PASS with HT autografts compared with BPTB autografts. Similarly, Kaarre et al^13^ found lower odds of not achieving the MIC with HT autografts in the short term. However, neither study included QT autografts.

Concomitant meniscal repair markedly increased the odds of subsequent meniscal procedure and reoperation due to extension deficit or pain and was associated with lower odds of achieving the PASS and higher odds of experiencing TF compared with no concomitant meniscal surgery. This aligns with previous studies reporting higher rates of subsequent meniscal procedures after meniscal repair performed concomitantly with ACLR.^19,24^ Interestingly, both meniscal repair and resection were associated with higher odds of achieving the MIC compared with no meniscal surgery, possibly reflecting more severe preoperative symptoms, allowing greater postoperative improvement and thereby increasing the likelihood of achieving the MIC. Regarding extension deficit or pain, previous studies have reported increased odds of reoperations for arthrofibrosis and cyclops lesions, both of which may cause extension deficit or pain, after ACLR with meniscal repair.^2,8,10,16^ Proposed explanations for this association include stricter range of motion restrictions after meniscal repair^2^ and the theoretical impact of the repair implant itself, which sits outside the capsule and is tightened to support the meniscus, potentially reducing knee joint mobility.^8^ Regarding age, patients aged <20 years had significantly superior outcomes in the PASS and TF, despite similar objective outcomes, likely reflecting greater healing capacity, higher activity levels and/or motivation to return to sports, and fewer pre-existing joint changes in younger patients.

Finally, the QT group experienced significantly more concomitant meniscal and cartilage injuries than the HT and BPTB groups at baseline. However, this did not translate into higher rates of reoperations for meniscal or cartilage injuries in the QT group at 2-year follow-up, aligning with regression analysis showing no graft-specific differences. Thus, increased reoperation rates for QT autografts appear driven by extension deficit or pain rather than concomitant injury severity.

The current findings have several important clinical implications. First, surgeons should be aware that QT autografts, although increasingly used, may carry an elevated risk of early complications and inferior short-term subjective outcomes. Second, careful attention to graft diameter and notch dimensions, particularly in female patients, might mitigate the risk of extension deficit or pain. Finally, preoperative counseling should address graft-specific risk profiles demonstrated in this study to help patients make informed decisions about graft selection.

This study has several strengths. Few prior studies have examined nonrevision reoperations after primary ACLR across QT, HT, and BPTB autografts in large cohorts. The use of QT autografts has increased in popularity in recent years, but several previous studies have either excluded them or lacked direct comparisons across graft types. In contrast, this study comprised over 800 ACLR procedures with QT grafts, alongside large sample sizes in the HT and BPTB groups in the main analysis, enhancing reliability and generalizability. Additionally, complementary measures such as MIC, PASS, and TF were used rather than only raw KOOS subscale scores, providing a more nuanced and clinically meaningful assessment of patient-reported outcomes. Lastly, all surgical procedures were performed at a single center using standardized surgical techniques and postoperative rehabilitation protocols, enhancing internal validity.

### Limitations

This study has several limitations. First, the retrospective design provides a lower level of evidence than prospective studies and is subject to potential selection, information, and unmeasured confounding biases. Second, PROM data were incomplete at 2 years, which may introduce response bias, although prior studies have indicated that nonresponders do not differ significantly from responders regarding preoperative KOOS scores.^11,20^ Third, the rationale underlying graft selection was not recorded, allowing possible systematic differences between graft groups that could affect outcomes, independent of graft type. The HT autograft was used considerably more frequently than both the QT and BPTB autografts, which may contribute to greater surgical experience and technical proficiency. Furthermore, BPTB graft sizing is based on the bone block rather than the graft itself, which overestimates the true graft diameter. Fourth, variations in surgical technique, graft preparation, or rehabilitation adherence were not accounted for and may influence postoperative outcomes. Additionally, although patients were followed postoperatively at the clinic for approximately 12 months, reoperations performed outside the institution may have been missed, potentially underestimating the true reoperation rate. However, patients could contact the clinic directly when needed during the first 12 postoperative months, likely mitigating this limitation.

## Conclusion

QT autografts were associated with significantly higher reoperation rates for extension deficit or pain and inferior PROM scores compared with HT autografts at 2 years after primary ACLR.
